# A Copernicus pipeline to create a highly resolved land cover map for modelling urban biodiversity in European cities

**DOI:** 10.1016/j.mex.2025.103415

**Published:** 2025-06-10

**Authors:** Meret Pundsack, Lisa Merkens, Wolfgang W. Weisser, Anne Mimet

**Affiliations:** a Terrestrial Ecology Research Group, Department of Life Science Systems, School of Life Sciences Weihenstephan, Technical University of Munich, Hans-Carl-von-Carlowitz-Platz 2, D-85354 Freising, Germany; b Laboratoire BiodivAG, DEP ENS SCIENCES Biologie, UFR SCIENCES, Université d'Angers, 2 Boulevard de Lavoisier 49045 ANGERS CEDEX 01, Germany

**Keywords:** Copernicus spatial data, Urban ecology, Urban biodiversity, Ecological modelling, Connectivity modelling, Landscape connectivity, Ecological connectivity, Urban Atlas, Copernicus pipeline for European urban land cover maps

## Abstract

Ecological models can provide planners with important information to integrate the needs of animals and plants into urban planning processes, thereby contributing to improving biodiversity and human well-being in cities. These models require urban land cover maps with high spatial (1–10 m) and thematic resolution (e.g., discriminating trees from shrubs). The generalization of the models relies on the widespread availability of similar land cover maps. While the EU’s Copernicus program is a first step, a highly resolved map with European extent is still lacking. We demonstrate how to leverage Copernicus geospatial thematic datasets to create a unified land cover map for ecological analyses in cities. A processing pipeline for a land cover map depicting the land covers impacting species occurrence at a high spatial resolution is described. Based on a literature review, we first define criteria for such a land cover map. We then

• Identify suitable Copernicus datasets,
• Combine Copernicus layers into a land cover map with high spatial and thematic resolution,
• Perform an accuracy assessment on the land cover map to ensure sufficient quality.

By making standardized land cover maps for urban ecological modelling more accessible, this methodology contributes to the mainstreaming of data-driven ecological models in urban planning.

## Graphical abstract

**Figure:**
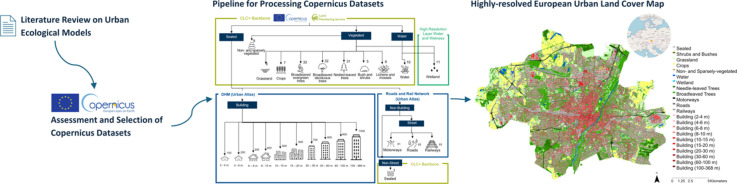
Process summary underlying the development of the Copernicus pipeline to create a highly resolved land cover map.

Specifications table

**Table:** 

Subject area:	*Environmental Science*
More specific subject area:	Ecological Modelling in Urban Areas
Name of your method:	Copernicus pipeline for European urban land cover maps
Name and reference of original method:	NA
Resource availability:	Copernicus datasets: freely available from - https://land.copernicus.eu/en/products/clc-backbone/clc-backbone-2021 - https://land.copernicus.eu/en/products/urban-atlas/building-height-2012 - https://land.copernicus.eu/en/products/urban-atlas/urban-atlas-2018 - https://land.copernicus.eu/en/products/high-resolution-layer-water-and-wetness/water-and-wetness-status-2018 R programming language: https://cran.r-project.org/terra package for R: https://cran.r-project.org/web/packages/terra/index.htmlsf package for R: https://cran.r-project.org/web/packages/sf/index.html

## Background

Planners increasingly aim to include the requirements of plants and animals in urban planning processes to make cities more liveable and biodiverse [1]. Predictive ecological models can inform planners by, for example, quantifying the amount of required resources, assessing their connectivity or evaluating species’ mortality. To account for the small-scale variations in the urban environment, such predictive modelling requires geodata at a high spatial and thematic resolution [2]. Following the Copernicus program, we define high spatial resolution as 3 to 10 m cell size and very high spatial resolution as below 3 m. A high thematic resolution is given when a land cover classification goes beyond broad or binary classes and often requires additional data sources or processing steps beyond raw images to, for example, distinguish between vegetation types (e.g. grass and trees) or sealed area types (e.g. various building height classes and streets). Thus, most urban ecological models use maps with small cell sizes detailing vegetation types serving as food, shelter or nesting sites, water bodies providing drinking water and habitat, and uninhabitable or disturbing spaces like built-up surfaces or traffic infrastructure [3,4].

To create such land cover maps, spatial information is mainly collected from regional data sources such as city administrations (e.g., [5]) and/or states (e.g., [6]), with different maps typically being combined (e.g., [2,6]). For example, App et al. [7] fused a 20 cm vegetation height raster with cadastre data to create a 2 m map detailing 20 land cover classes. Assembling geospatial data from multiple sources is labour-intensive, often requiring extensive preprocessing by experts (e.g., [5,7]). Moreover, while data on traffic infrastructure is widely available, thematically and spatially highly resolved vegetation maps are rare and often replaced by garden and public green plans (e.g., [7]). However, the administrative property of urban green spaces does not reflect actual vegetation structure and its functionality for biodiversity. Thus, standardized maps of urban green compatible with information on building- and traffic infrastructure are required.

Land cover maps compiled from multiple datasets often become incomparable even within countries because municipalities and states use different remote sensing technologies, prioritizing availability over ecological considerations for data selection. Efforts to employ a model created for Zurich to German and French cities [2,6,7] furthermore testify that using geospatial data from different sources complicates comparisons and applications between cities. Since the acquired land cover maps varied substantially between countries, model parameters were transferred from one map to another despite differing land cover classes [2,6,7], questioning the validity of the model transfer. Hence, to better incorporate ecological models into urban planning, there is a need for standardised, highly resolved land cover maps that can be easily replicated from one city to another beyond country boundaries. Cross-continental ecological research in many cities would be facilitated by such land cover maps, which would help identify general patterns in urban ecology.

The EU’s Earth Observation Programme Copernicus provides satellite data for improved analysis and management of the environment [8]. Copernicus datasets represent a major step towards standardized continent-wide open-access urban land cover data, providing 3-yearly updated spatial datasets at 10 m spatial resolution for European cities [9]. Since prior work by Oliveira et al. [10] demonstrates the value of Copernicus spatial data to unifying urban climate classes in Europe, we focus on Copernicus geodata to create a highly resolved urban land cover map. However, individual Copernicus datasets do not combine ecological information on vegetation with uninhabitable spaces such as roads and buildings. Thus, existing Copernicus datasets require processing, yet no processing pipeline exists.

To our knowledge, this is the first study to develop a standardised, reproducible processing pipeline for Copernicus data to integrate both ecological (e.g. vegetation types) and anthropogenic (e.g. roads, buildings) land covers in a highly resolved single land cover map. This study establishes standardised criteria for spatial and thematic data resolution in urban ecological modelling studies. While prior research has included individual Copernicus datasets to account for either natural or anthropogenic land covers, we provide a detailed pipeline for producing a multifaceted land cover map that is valuable for studies focusing on the natural and anthropogenic environment, likewise. The pipeline 1) integrates ecological and anthropogenic landscape covers, 2) provides explicit and replicable data resolution standards, and 3) produces a land cover map suitable for pan-European ecological studies.

To concretize the criteria for spatial data collection and processing, we will use the example of urban connectivity modelling. We focus on connectivity models because they rely heavily on land cover maps for depicting resources and barriers and thus require high thematic and spatial resolution [3]. Since these models demand a detailed depiction of natural and anthropogenic land covers, a land cover map fulfilling the requirements for ecological connectivity models is also suitable for a wide range of other ecological models in cities.

## Method details

**Fig. 1:**
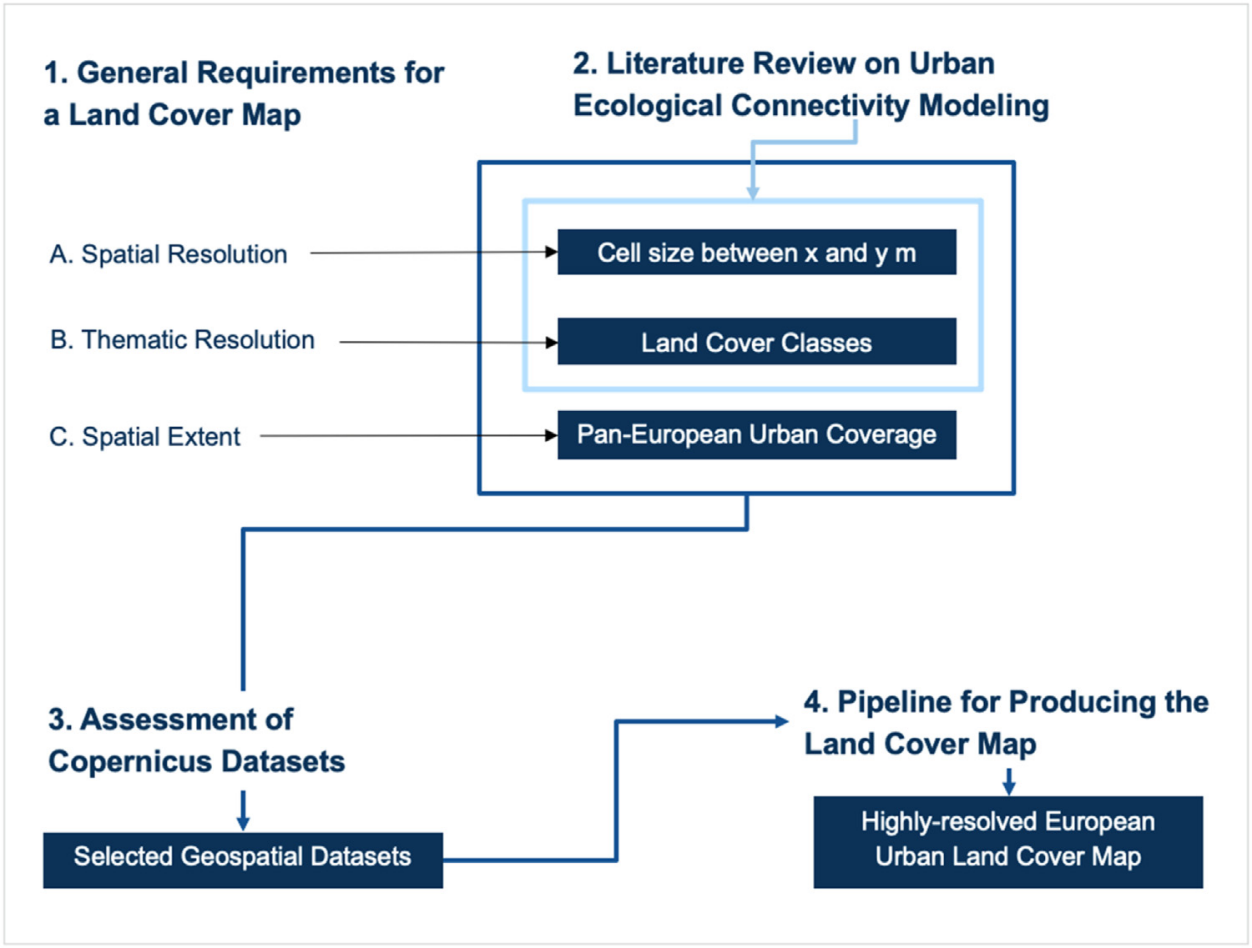
Overview of the methodological steps taken to derive criteria for European land cover map, identify suitable spatial datasets and process them in a highly-resolved European urban land cover map.

We applied four steps to derive a processing pipeline to create a unified, highly-resolved European urban land cover map for ecological modelling in cities (Fig. 1):

1. definition of general requirements for a land cover map regarding spatial and thematic resolution, coverage and extent,
2. literature mini-review to concretize criteria defined before, especially which thematic and spatial resolution is available and required,
3. assessment and selection of open-access Copernicus datasets and
4. development of the pipeline for producing a Copernicus land cover map.

### General requirements for a highly resolved land cover map for European cities

The aim of this study is to develop a pipeline for a pan-European land cover map for urban ecological studies. Since Copernicus provides a multitude of thematic geospatial datasets, the production of only one map requires selection from a variety of possible input data. In order to identify a subset of suitable data, we first defined criteria for land cover maps and then developed a data processing pipeline. The suitable data must match the three main requirements, which regard (1) spatial resolution, (2) thematic resolution and (3) spatial extent and coverage.

Land cover maps used for urban ecological modelling differ in their thematic resolution, i.e. the land cover classes that they depict, and their spatial resolution, i.e. the size of the map’s grid cells (e.g. [2,[5], [6], [7]]). This is due to the strong dependence of the required spatial and thematic resolution on the ecology of the target species, the difference between cities and countries regarding the availability of high-quality datasets, computing power and limitations linked to the chosen modelling approach. The size of the modelled animal heavily impacts the spatial resolution required because its body mass affects the spatial scale at which it perceives the environment [4], whereas the thematic resolution needs to reflect the habitat(s), resources, and the barriers that an animal perceives [2,4]. However, the thematic resolution is constrained by the spatial resolution of the imagery, which determines the level of detail that can be distinguished using image interpretation methods. The latter is typically limited by the characteristics of the sensor capturing the imagery. In the case of spaceborne sensors, a higher satellite altitude allows for wider area coverage but results in larger pixels – a trade-off inherent to the mission’s purpose. Moreover, a high spatial resolution inflates the computational power required to perform the modelling, with dynamic ecological models taking even more computer resources than statistical approaches. Hence, the thematic and spatial resolution are always in trade-off with the geographical extent at which the data can be assembled and the computing power available to run spatial analyses.

Regarding the spatial extent, we decided to work at the European scale because restoring urban biodiversity is one of the EU’s main biodiversity goals until 2030 [11]. The example of urban ecological models developed for one city and then applied to two other European cities demonstrates the potential that fully transferable models could have if they are based on the same input data [2,6,7]. If fully transferable, a model developed in one European city could inform urban planning in many others and thereby boost the use of ecological models in urban planning. However, the aim to create a pan-European urban map does not suffice to determine the extent required from the input datasets. The availability of pan-European data depends on the institution providing it, whereas the definition of cities varies substantially between countries [12].

We concluded that, despite aiming for a pan-European urban land cover map suitable for many species, we had to define minimum criteria for spatial and thematic resolution and spatial extent. Therefore, we reviewed the literature to gain an overview of urban land cover classes used for urban ecological modelling, data availability, extent, coverage and spatial resolution. Since it was beyond the scope of this study to perform a comprehensive literature review on the land cover maps used in all types of urban ecological modelling studies, we decided to focus on ecological connectivity models in cities. These analyses are strongly reliant on spatially and thematically highly resolving land cover maps and require increasing computing power at finer spatial resolution [3].

Thus, ecological connectivity models have a wide set of requirements regarding the land cover maps used. If the requirements are fulfilled for those models, the resulting land cover maps are also informative for many other modelling studies. We concluded the literature mini-review with a definition of criteria for a pan-European land cover map useful for ecological modelling applicable between major European cities. We specified the criteria and their importance to make a clear decision in case of one dataset complying with one criterion but not another. Applying the criteria and their importance defined before, we then assessed the suitability of different datasets provided by Copernicus. Finally, we describe a pipeline that processes the identified Copernicus datasets to generate a pan-European unified land cover map.

### Literature mini-review on land cover maps for ecological connectivity modelling in cities

Regarding the land cover maps used in ecological connectivity models in cities, we performed a review of 41 scientific publications (Table 1). The literature included in the mini-review was assembled from a collection of peer-reviewed studies collected over several years of research in the field of urban connectivity modelling. While this does not constitute a systematic review, it represents a broad and methodologically sound sample of urban connectivity modelling studies from which consistent information on the use of land cover maps for connectivity modelling could be extracted. We selected peer-reviewed publications using raster land cover maps to perform resistance-based connectivity modelling in cities based on least-cost path, circuit and graph theory modelling approaches. In these publications, the land cover maps inform about the locations of the nodes of the connectivity model, i.e. of locations that attract an animal, as well as the resistance of land covers to movement. We extracted information on the administrative level at which the data was gathered (e.g. local unit such as a municipality, regional unit such as a federal state like Bavaria, country such as Germany, and continent such as Europe) as well as the spatial resolution at which the connectivity was modelled. Moreover, we evaluated whether the urban study area was defined based on administrative boundaries, functional definitions of cities or other considerations. Finally, we gathered the map’s land cover classes. We counted the publications using a specific land cover class and summarized synonymous land cover classes. Not every publication clearly stated how the land cover map was retrieved or at what resolution the modelling was performed. However, if the publication still detailed all land covers considered in the connectivity modelling, we also retained manuscripts from which information on resolution and/or administrative level of data could not be extracted. Moreover, we established and visualized a hierarchy to depict that some land cover maps are more finely resolving a land cover class that was also present in other land cover maps, e.g. the class street in one study was divided into primary, secondary, and tertiary road in another publication. Table 1 provides an overview of all reviewed publications.

**Table 1:** Overview of publications reviewed to enhance understanding of the administrative data sources, spatial resolution, and land cover types in resistance-based connectivity studies in cities. Entries are sorted from very high to coarse spatial resolution. Empty cells indicate that the information could not be derived from the respective manuscript and its supplementary information If several administrative data sources are mentioned, this indicates that the study obtained and combined spatial data from multiple administrative levels (see Appendix A for an overview of the publications reviewed).

Study	Year	Geographic location	Species / taxon	Modelling approach	Spatial resolution [m]	Administrative data source	Application domain
App et al. (2022)	2022	Braunschweig, Germany	Hedgehog	Circuit theory	2	Local unit	Urban planning
Beaujean et al. (2021)	2021	Two parts of Liège, Belgium	Natterjack toad	Least-cost paths, circuit theory	2	Local unit, Regional unit	Urban planning
Braaker et al. (2014)	2014	Zurich, Switzerland	Hedgehog	Circuit theory	2	Local unit, Country,	Urban ecology
Ersoy et al. (2019)	2019	Sheffield, England	10 vertebrate species	Least-cost corridors	2	Country	Urban planning
Verbeylen et al. (2003)	2003	Brussels, Belgium	Red squirrel	Least-cost paths	2	Country	Landscape ecology
Egerer et al. (2020)	2020	Baltimore/Chicago/New York, USA	General ecological processes	Circuit theory	3	Regional unit, Country	Urban planning
Morin et al. (2022)	2022	Châtellerault/Niort/Poitiers, France	General ecological processes	Graph theory	3	Regional unit, Country	Remote sensing
Kosma et al. (2023)	2023	Parts of Jyväskylä, Finland	Flying squirrel	Graph theory	4	Country, Continent	Biodiversity offsetting
Bhakti et al. (2021)	2021	Part of Ouro Preto, Brazil	Forest birds	Least-cost corridors	5	Local unit	Urban planning
Grafius et al. (2017)	2017	Milton Keynes/Luton/Bedford, UK	Blue tit, great tit	Circuit theory	5	Country	Urban ecology
Graviola et al. (2022)	2022	Rio Claro, Brazil	Bird	Least-cost corridors	5	Local unit, Regional unit	Urban planning
Balbi et al. (2019)	2019	Rennes, France	Hedgehog	Least-cost paths	5	Country, Continent	Urban planning
Balbi et al. (2021)	2021	Rennes/Lens, France	Moths, birds	Least-cost paths	5	Country, Continent	Urban planning
Kong et al. (2021)	2021	Part of Nanjing, China	General ecological processes	Circuit theory	10	Local unit	Conservation biology
Magle et al. (2009)	2009	Denver and surroundings, USA	Prairie dog	Least-cost paths	10	Local unit, Regional unit	Landscape ecology
Braaker et al. (2017)	2017	Zurich, Switzerland	Hedgehog	Circuit theory, least-cost paths	10	Local unit, Country	Landscape genetics
Tannier et al. (2016)	2016	Besançon and its urban region, France	Forest mammals	Graph theory	10	Local unit, Country	Urban planning
Matos et al. (2019)	2019	Central England, UK	Great crested newt	Graph theory	10	Regional unit, Country	Conservation biology
Molné et al. (2023)	2023	Zurich canton/Aargau canton, Switzerland	4 amphibian species	Graph theory, circuit theory	10	Regional unit, Country	Conservation biology
Driezen et al. (2007)	2007	Oxford/Rousham/Sandford/Wilcote/ Eynsham, England	Hedgehog	Least-cost paths	10	Country	Ecological modelling
Han & Keeffe (2019)	2019	Greater Manchester, England	Trees	Least-cost paths	10	Country	Climate change mitigation
Mimet et al. (2020)	2020	Paris, France	Common pipistrelle	Circuit theory	20	Local unit	Urban planning
Tarabon et al. (2020)	2020	Toulouse conurbation, France	20 species	Graph theory	20	Regional unit, Country, Continent	Urban planning
Beninde et al. (2016)	2016	Trier, Germany	Common wall lizard	Circuit theory	25	Local unit	Landscape genetics
Marulli & Mallarach (2005)	2005	Barcelona Metropolitan Area, Spain	General ecological processes	Least-cost paths	25	Regional unit	Urban planning
Zetterberg et al. (2010)	2010	Stockholm, Sweden	Common toad	Graph theory	30	Local unit, Country	Landscape ecology
Liu et al. (2022)	2022	Beijing, China	General ecological processes	Graph theory, circuit theory	30	Local unit, Continent	Urban planning
Yu et al. (2012)	2012	Shenzhen, China	General ecological processes	Graph theory	30	Local unit, Continent	Ecological restoration
Hou et al. (2021)	2021	Fenhe River Basin, China	General ecological processes	Least-cost paths	30	Regional unit	Urban planning
LaPoint et al. (2013)	2013	Around Albany, USA	Fisher	Least-cost paths, circuit theory	30	Regional unit, Country	Urban ecology
Tang et al. (2020)	2020	Wuhan, China	General ecological processes	Least-cost paths	30	Regional unit, Country	
Zhang et al. (2024)	2024	Hangzhou Bay, China	General ecological processes	Circuit theory	30	Country	Landscape planning
Huang et al. (2021)	2021	Wuhan, China	General ecological processes	Least-cost corridors	30	Continent	Landscape planning
Miao et al. (2019)	2019	Wuhan, China		Graph theory	30	Continent	Urban planning
Nor et al. (2017)	2017	Kuala Lumpur, Malaysia/Jakarta, Indonesia/Metro Manila, Philippines	Eurasian tree sparrow, yellow-vented bulbul	Least-cost path	30	Continent	Urban planning
Shen et al. (2023)	2023	Suzhou/Jiaxing/Huzhou, China	General ecological processes	Graph theory	30	Continent	Urban planning
Zhao et al. (2019)	2019	Tianjin City, China	General ecological processes	Graph theory	30	Continent	Urban planning
Shimazaki et al. (2016)	2016	Sapporo/Ebetsu/Ishikari, Japan	6 bird species	Circuit theory	50	Country	Urban planning
Laforge et al. (2019)	2019	Lille conurbation, France	Bats	Least-cost paths	250	Country	Urban ecology
Grabow et al. (2022)	2022	Berlin, Germany	Red squirrel	Circuit theory	500	Continent	Urban ecology
Lee et al. (2022)	2022	Calgary, Canada	Wood frog, Boreal Chorus frog	Circuit theory		Local unit	Urban planning

**Fig. 2:**
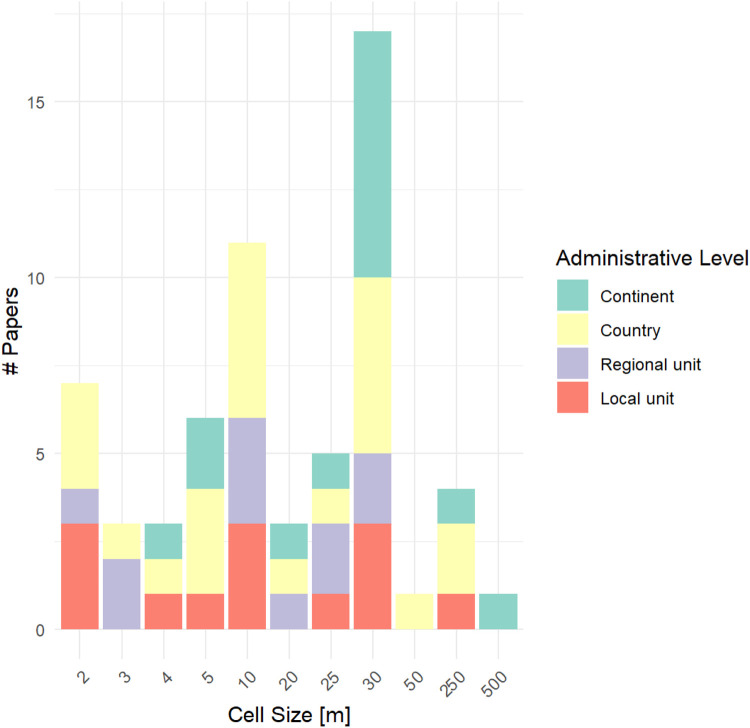
Number of papers in which a certain cell size (in m) was used for the land cover map that was inputted into a resistance-based connectivity model. The colours indicate at which administrative level the geodata used to create the land cover map were created and gathered.

The assessed publications differed in the scale and resolution of application; some strictly referred to only one animal species, whereas others assessed the connectivity of urban landscapes to more general ecological processes (Table 1). We found that most publications gathered geoinformation at the country level, whereas continental, regional (e.g. federal state) and local (e.g. muncipality) data sources were relatively equally represented. Several publications also used geospatial data from different administrative levels and combined them (Table 1). Moreover, most publications modelled ecological connectivity at a 30 m resolution, whereas the second most frequently represented resolution was 10 m (Fig. 2). Nevertheless, in 18 publications, resistance-based connectivity was analysed using a high spatial resolution below 10 m cell size. We also noticed that a continent-wide acquisition of geospatial data was often related to a resolution of 30 m (Fig. 2) because the land cover map was generated using Landsat data. Land cover maps created from Landsat typically only included a few land cover classes while connectivity modelling was then applied to a regional scale around the focal city to assess a city’s general conductivity. The spatially more finely resolved studies typically acquired geospatial data from federal or national institutions (country level), regional units or local units (Fig. 2). They provided a higher thematic resolution of urban land cover classes and focused on within-city movement.

**Fig. 3:**
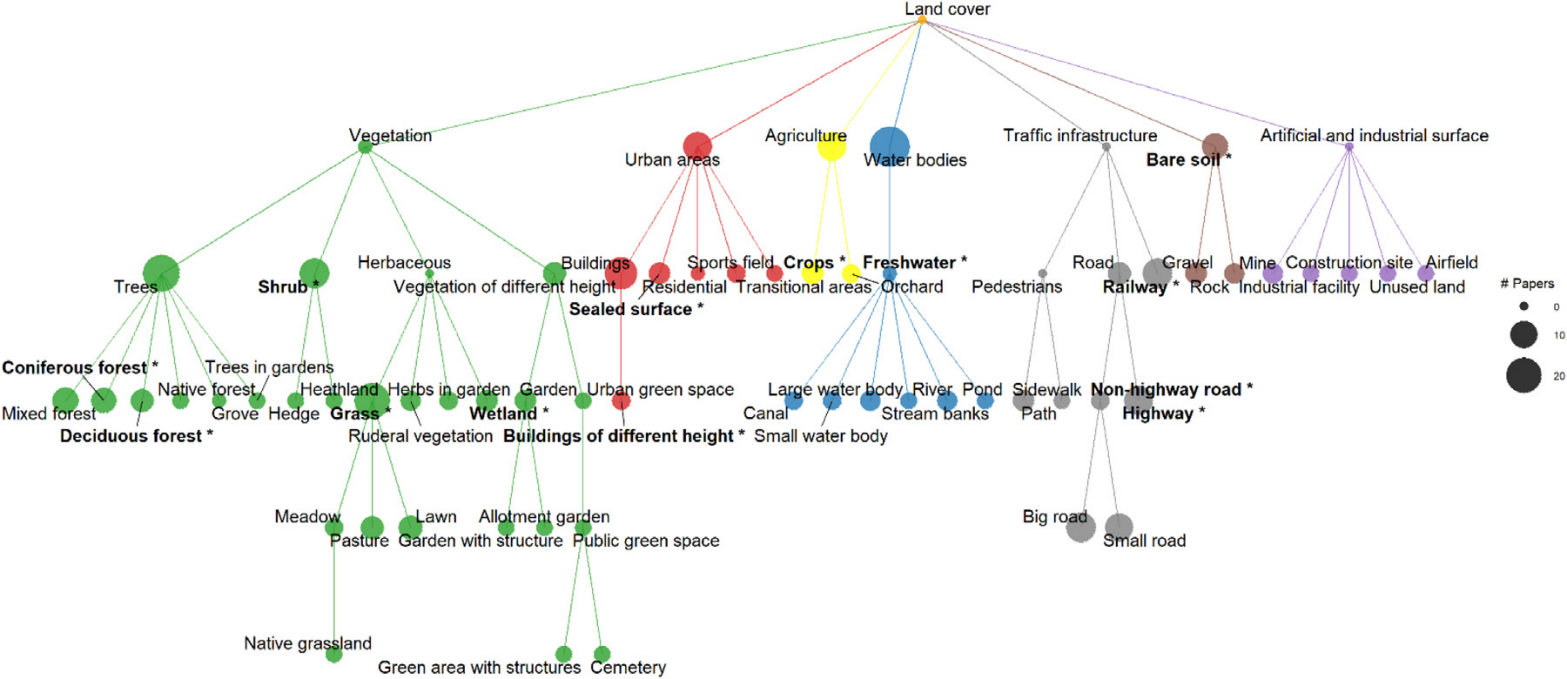
Dendrogram showing the hierarchy and number of land cover classes in the evaluated publications. The size of the dot per land cover class indicates the number of papers in which this specific land cover class at the respective hierarchy was mentioned. Bold writing with * indicates the hierarchy level that was available from the Copernicus land monitoring service and that is represented in Copernicus land cover map. Terminal nodes with <2 occurrences in publications were dropped to improve the visibility of the dendrogram.

Concerning the thematic resolution, we furthermore found that all screened publications differentiated vegetation from urban areas, agriculture, water bodies, and traffic infrastructure, whereas information on bare soil and artificial and industrial surfaces was included less often (Fig. 3). Vegetation was typically divided into trees, shrubs and different types of herbaceous vegetation, while many publications even identified more specific vegetation cover classes (Fig. 3). Generally, urban areas were either not differentiated (mostly when Landsat input data was used) or divided into buildings and sealed surfaces (Fig. 3). Within the agriculture land cover class, crops were often distinguished, whereas only few publications resolved the water body class more finely (Fig. 3). Within the class of traffic infrastructure, many publications distinguished railways from roads and classified roads as highways, smaller and bigger roads (Fig. 3). Thematically very fine resolved land cover classes, such as different types of grasslands or water bodies, were rarely represented in the literature and typically reflected the specificities of the used geospatial data that was often combined with administrative information provided by the municipality.

In most studies, the definition of the urban study area was based on the administrative boundaries of the city or the region, although few studies focused only on parts of the city. Some studies also used a more functional definition of the study area than the administrative boundary (Table 1). This was typically the case if dense settlements surpassed the city boundary, for example, in France. However, no study used a more general approach to define the boundary of the study area, such as the Functional Urban Area approach or the EU functional cities that typically include areas beyond the administrative boundary and use population density to define urban areas [12].

Many publications used data obtained at a continental scale, but this was then related to coarser spatial and thematic resolutions, whereas locally obtained data was typically used in more fine-scaled modelling. While using a coarse land cover map is valid when assessing ecology at a regional scale, it is not suitable to depict small-scale processes, such as which land cover classes support animal movement between urban green spaces (e.g. [2,5]). We conclude that while many connectivity studies employ spatial data with a grid cell size of 30 m, many others also successfully worked at a finer resolution when data were assembled from national, state, or municipal sources. However, since these datasets are not unified within Europe (and often not even within a country), they cannot support the application and comparison of connectivity models between European cities. Regarding the land cover classes, we found that there was a general agreement on discerning land cover classes of different vegetation types, buildings, roads and sealed surfaces within urban areas, water bodies, agriculture and bare soil. Nevertheless, the thematic resolution differed strongly between studies. Defining the study area based on the administrative boundaries of the cities was most frequently represented in the literature. However, modelling results could be difficult to compare and transfer between countries that employ different approaches to defining city boundaries.

Based on the literature review and our intent to provide input available for pan-European urban ecological studies, we defined concrete criteria for (1) spatial resolution, (2) thematic resolution, and (3) data extent and coverage. Moreover, to make unequivocal decisions in case that some criteria were met and others were not, we ranked the criteria by importance.

(1) **Spatial resolution:** The land cover map should have a high- to -very high spatial resolution between 1 and 10 m to account for the small scale at which the urban environment varies drastically. To reduce computational requirements, spatial resolution can always be reduced to a coarser resolution if the animal’s ecology does not require a small pixel size.
(2) **Thematic resolution:** We extracted the most frequently represented land cover classes from the literature that should minimally be included in the land cover map:
  1. **Vegetated and natural land cover classes**: Trees, Grass, Shrubs, Bare Soil, Agriculture, Water bodies
  2. **Anthropogenic land cover classes**: Sealed surfaces (no roads, not built-up), Buildings, Roads, Railway
  If available, a more granular thematic resolution is preferable. Land cover classes can be aggregated later if the animal’s ecology does not require a high thematic granularity.
(3) **Spatial coverage and extent:** The geographic coverage of the land cover map should include as many European cities as possible, at least all major cities within the EU. The EU provides a functional definition of cities, where urban areas are specified as areas with >1500 inhabitants/km^2^ and >50,000 inhabitants in total [13]. The EU functional city boundaries, defined as functional urban area are typically larger than the administrative cities, but they belong to a similar spatial scale. The approach to model only within a city’s administrative boundaries is self-evident when providing recommendations for planners, we however recommend considering the more functional definition by the EU that is especially applicable to urban areas where only parts of an urban agglomeration form the administrative city.

Even when working with spatially highly resolved data, modelling at this spatial scale is computationally feasible. We conclude that regarding the geographic coverage, the dataset should at least encompass all functional European cities to incorporate all major European cities while leaving the decision of spatial boundary definition to the user according to the needs of their modelling endeavour. If the spatial extent of a map is determined by the functional city, then the full administrative area is covered by the map [13].

**Evaluation metrics:** Spatial and thematic resolution determine the suitability of land cover maps for ecological modelling studies. Therefore, we decided that criteria 1) high spatial resolution (1 – 10 m) and 2) high thematic resolution are of overarching importance. Copernicus datasets not meeting these criteria were excluded immediately. Regarding the spatial extent and coverage, the use of Copernicus datasets led to the pan-European coverage always being fulfilled. However, we allowed for some inconsistencies in the coverage of functional cities across datasets if the datasets covered all major urban areas in Europe. Hence, criterion 1) spatial resolution and criterion 2) thematic resolution were of highest importance, whereas criterion 3) spatial extent and coverage was not employed strictly.

Update frequency and classification accuracy were not used as hard selection criteria since we conducted an independent accuracy assessment of the full land cover map against recent orthophotos.

### Selection and evaluation of Copernicus datasets

To evaluate the suitability of Copernicus datasets for creating a European urban land cover map, we applied the three previously defined criteria 1) high spatial resolution, 2) high thematic resolution and 3) pan-European urban extent and coverage. As outlined before, we compromised on the definition of the spatial extent and coverage of European cities if criterion 1) and 2) were met. Based on the three identified criteria, we reviewed the different available processed spatial datasets provided by the Copernicus program. The selection procedure incorporated the consideration of various spatial datasets. We will describe the datasets available and thereby select the datasets suitable for our intent to create a consistent land cover map applicable across European cities.

The overall purpose of the European Union’s Copernicus is to provide free access to information services in accordance with the INSPIRE (Infrastructure for Spatial Information in the European Community) Directive by the EU, aspiring for conformity and accessibility of spatial datasets for all EU member states [14]. Reuse for scientific purposes of their datasets is highly encouraged while pan-European coverage is ensured, highlighting the relevance of the Copernicus spatial datasets for this research. Within the Copernicus Program, pre-processed and-/or classified spatial data such as the *Global Human Settlement Layer (GHSL)*, the *CLC+ Backbone raster layer* and the *Tree Cover Density (TCD)* raster are released [15].

An individual Copernicus dataset was not found to be sufficient as a singular data input since datasets often focus on a particular aspect of the landscape, such as vegetation or water, and, therefore, no individual dataset complied with the desired high thematic resolution. The *CLC+ Backbone* dataset, for example, focuses on discriminating between different vegetation types, whereas the *GHSL* describes the built-up structure while only indicating NDVI (Normalized Difference Vegetation Index) values for vegetation land cover [14,16]. Thus, multiple spatial datasets had to be combined, making prior modifications mandatory.

Each classification dataset with a spatial resolution coarser than 10 m was excluded applying the 1st criterion on spatial resolution. Since Copernicus provides data for 39 European countries including UK and Turkey, data from Copernicus generally complies with the pan-European coverage.

The *CLC+ Backbone 2021* dataset, which has a 10 m spatial resolution and a three-yearly update frequency, provides a land cover inventory for Europe aimed at environmental applications and policy making. This dataset was selected as the foundation for the land cover map since it distinguishes the land surface into 11 distinct classes, focusing on discriminating between different vegetation types [14]. Vegetation is divided into woody – broadleaved deciduous trees, woody – broadleaved evergreen trees, woody – needle-leaved trees, periodically herbaceous (arable land), permanently herbaceous (grassland), low-growing woody plants (bushes and shrubs), and lichens and mosses. The remaining classes are the following: sealed, water, as well as non- and sparsely vegetated. One major advantage of the *CLC+ Backbone* dataset is the usage of auxiliary datasets by the European Environment Agency (EEA) for postprocessing [14]. To reduce the omission of urban trees for example, the EEA utilized the *Tree Cover Density High-Resolution Layer 2018* with a spatial resolution of 5 m while, for reducing the omission of herbaceous vegetation on roads, they utilized the *High-Resolution Grassland Layer 2021* with a 10 m spatial resolution. The accuracy of the *CLC+ Backbone* dataset was assessed to be above 90 %, representing a solid depiction of the urban landscape at a 10 m resolution [17]. In addition to that, we employed the *High Resolution Layer Water and Wetness 2018* dataset to obtain additional information on wetlands [18]. This dataset, with a 10 m spatial resolution and a three-yearly update frequency, is primarily intended for applications in water management and related policies. Both datasets are available for the entire terrestrial European surface, including all cities.

To account for the effect that buildings and their different heights exhibit on urban biodiversity by, for example, altering the landscape resistance, an adequate description of buildings and their respective heights is additionally required [19]. We chose the building height classes of the *Urban Atlas Digital Height Model (DHM) 2012* spatial dataset with a 10 m spatial resolution and an update frequency based on demand for applications relating to urban areas from which we retrieved granular building height information [20]. To further distinguish the remaining sealed area into solely sealed areas and street or road infrastructure, spatial vector data released as part of Copernicus’ *Urban Atlas 2018* was once again considered, and the roads- and rail network classes were utilized [9,14]. The Copernicus *Urban Atlas 2018* vector data with a minimum mapping unit (MMU) of 2500 m^2^, a minimum mapping width (MMW) of 10 m, and an update frequency based on demand, was produced for applications relating to urban areas and is thus available for most European functional cities. For a few European functional cities, the *Urban Atlas Digital Height Model 2012* and the *Urban Atlas 2018* do not provide data. Thus, we accepted that not all European functional cities were represented in the two *Urban Atlas* datasets and compromised on the 3rd criterion since both *Urban Atlas* datasets contain unique thematic information. Table 2 shows the spatial datasets considered for the land cover map production in more detail and demonstrates that all land cover classes defined in the 2nd criterion could be differentiated by the selected Copernicus datasets.

**Table 2:** Copernicus spatial datasets considered for producing a European land cover map for urban ecological modelling. Thematic accuracy measures how accurately the classified land cover types represent the ground truth [21]. The blind approach, here shown in brackets, indicates the thematic accuracy conducted without prior knowledge of ground truth or later contextual correction.

Dataset	Land Cover Classes (Final classes in bold)	Land Cover Class Criterion fulfilled	Spatial extent	Spatial resolution [m] & Datatype	Input Data, Sensor	Thematic Accuracy (blind approach)	Included/ Excluded
**Included Datasets**							
**Vegetation and Natural Land Cover Focus**							
CLC+ Backbone 2021 (3-yearly update)	– Needle-leaved trees – Broadleaved trees – Grass – Shrubs – Agriculture – Lichens and mosses – Non and sparsely vegetated – Water bodies – Sealed	1) Trees 2) Grass 3) Shrubs 4) Water bodies 5) Agriculture 6) Bare soil 7) Sealed surfaces	Continuous European	10 m Raster	Sentinel-2 L2A	90 % (77.5 %)	Included, good description and high thematic resolution of vegetation
High Resolution Layer Water and Wetness 2018 (3-yearly update)	– Wetland		Continuous European	10 m Raster	Sentinel-1, Sentinel-2	80 % Permanent Wet (80 %)	Included, additional information on wetland
**Anthropogenic Land Cover Focus**							
Digital Height Model (DHM) – UrbanAtlas 2012 (Update as needed)	– **Buildings** (distinguished into 10 different classes from height of 2 - 368 m)	8) Buildings	785 European functional cities with > 50,000 inhabitants	10 m Raster	WorldView-1/2/3, GeoEye-01, Quickbird-01, Ikonos	Not available	Included, stringent identification of building structures
Roads and Rail Network (extracted from UrbanAtlas) 2018 (Update as needed)	– **Fast transit roads** – **Other roads** – **Railways**	9) Roads 10) Railways	788 European functional cities with > 50,000 inhabitants	Vector 2500 m^2^ min. mapping unit and 10 m min. mapping width (relevant classes rasterized 10 m)	Pleiades, KOMPSAT, Planet, SPOT6, SuperView	87.5 % (68.5 %)	Included, additional distinction between sealed land cover and road infrastructure
**Excluded Datasets**							
High Resolution Layer Small Woody Features 2018 (3-yearly update)	Small vegetation elements such as hedgerows and other woody features		Continuous European	5 m Raster	Pleiades 1A/1B, SuperView-1, KOMPSAT-3/3A, PlanetScope	94.09 % (Not available)	Excluded, little addition of vegetated patches to *CLC+ Backbone 2021*
GHS-BUILT-C R2023A (Irregular updates)	Building Height distinguished into Residential and Non-Residential Buildings		All global Functional Urban Areas	10 m Raster	Sentinel-2 Composite, ALOS Global Digital Surface Model, NASA Shuttle Radar Topography Mission	Not available	Excluded, no identification of stringent building blocks

### Pipeline for producing the land cover map

**Fig. 4:**
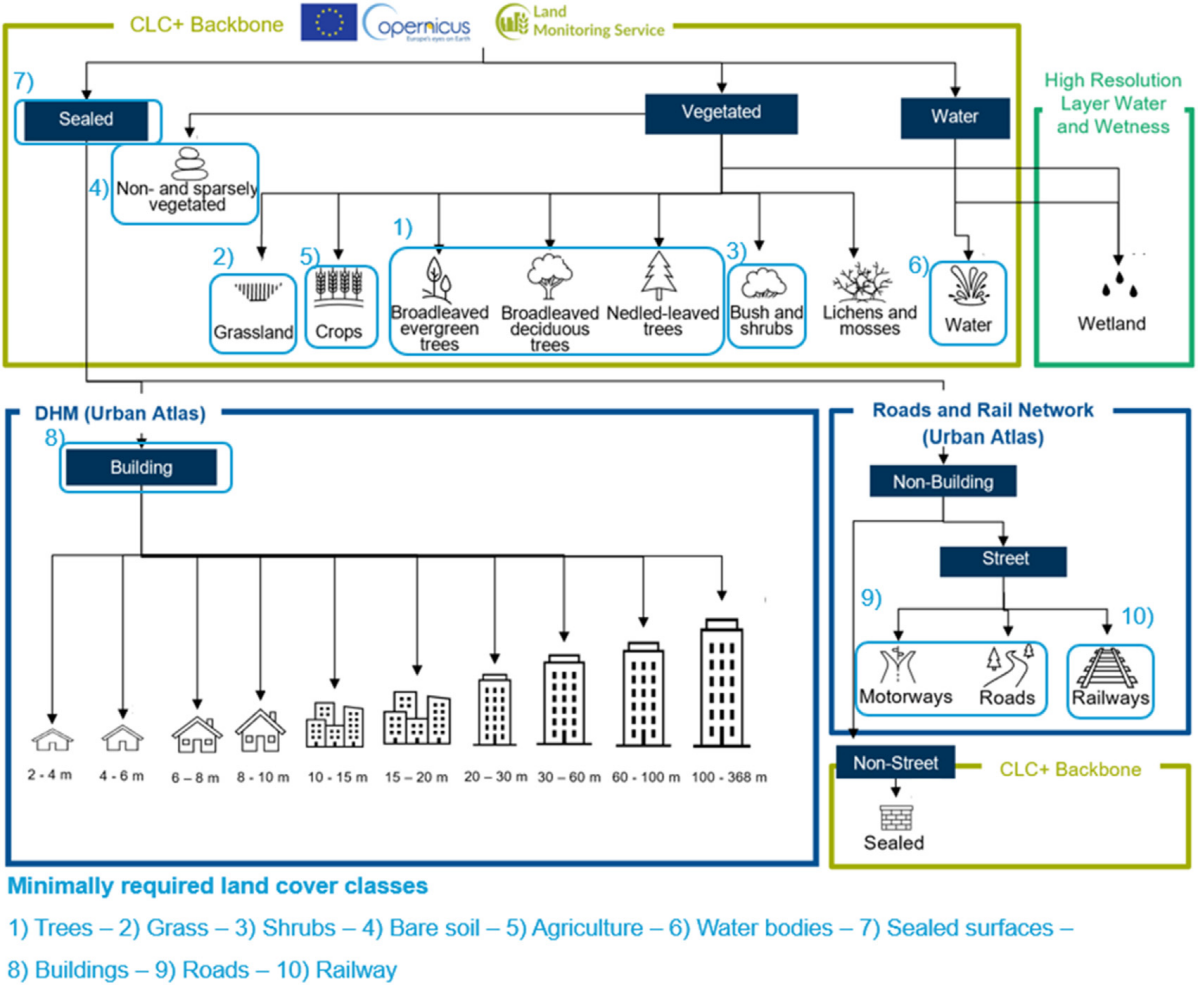
Layer stacking and reclassification procedure for land cover map production from Copernicus datasets: CLC+ Backbone 2021, High Resolution Layer Water and Wetness Status 2018, Digital Height Model (DHM) Urban Atlas and Roads and Rail Networks (extracted from the Urban Atlas 2018). Class indications with pictograms indicate final land cover classes. Boxes with light blue frames and numbers indicate which land cover classes represent the minimally required land cover classes identified in the literature review and that are also listed at the bottom.

The proposed pipeline integrates the selected datasets in a specific order with the CLC+ Backbone 2021 dataset to minimize conflicts between land cover classes (Fig. 4). The integration workflow uses R v.4.3.0 [22] making our pipeline easily applicable and reproducible for others.

As depicted in Fig. 4, the *CLC+ Backbone 2021* dataset represents the basis of the land cover map. The classification from *CLC+ Backbone 2021* groups land covers into three main categories: Sealed, Vegetated, and Water. Vegetated land is further distinguished into different vegetation types: Broadleaved trees, Needle-leaved trees, Grassland, Crops, Shrubs, Lichens and Mosses, Non- and sparsely vegetated. Water and Vegetation classes are further refined by overlaying this class with the second spatial dataset, the *High-Resolution Layer Water and Wetness* 2018 dataset, and reclassifying the land cover to Wetland (Permanent Wet) whenever identified. Thereby, the land cover class Wetland is added to the land cover map.

To further refine the classification of sealed areas and to incorporate crucial information on the height of buildings, we apply a conditional raster overlay for all pixels classified as sealed land cover to be replaced by the third spatial dataset, the *Digital Height Model (DHM)* of the *Urban Atlas 2018*. Thus, every *Sealed* pixel obtains either a specific height value or none assuming the absence of buildings in that cell. Due to the granular distinction of heights in the *DHM*, 10 distinctive classes are obtained (see Fig. 4).

Sealed land cover pixels without height being attributed from the DHM are considered *Non-built-up Spaces* and further classified by applying a conditional raster overlay with the aid of the third dataset, the *Roads and Rail Network of the Urban Atlas 2018* dataset. These classes were initially extracted from the *Urban Atlas* vector dataset, buffered by 7 m to ensure little information loss and rasterized by the maximum area cell assignment. This is done using the *terra* package's *rasterize* function [23]. The 7 m buffer, in combination with the maximum area cell assignment type, performed best at describing the streets and not losing land cover information from the *Urban Atlas 2018* dataset. We reclassify all remaining pixels in the *Non-built-up Spaces* as Sealed. Thereby, the land cover map consisting of 23 land cover classes is produced. To replicate the processing in any European city, an R-script accompanies this paper.

## Method validation

To demonstrate the validity of our approach, we acquired the required Copernicus data for the German municipality of Munich and applied the pipeline described before to create a land cover map of Munich. Additionally, an accuracy assessment was conducted to test the land cover map’s quality for detecting different land covers.

### Land cover map of Munich

**Fig. 5:**
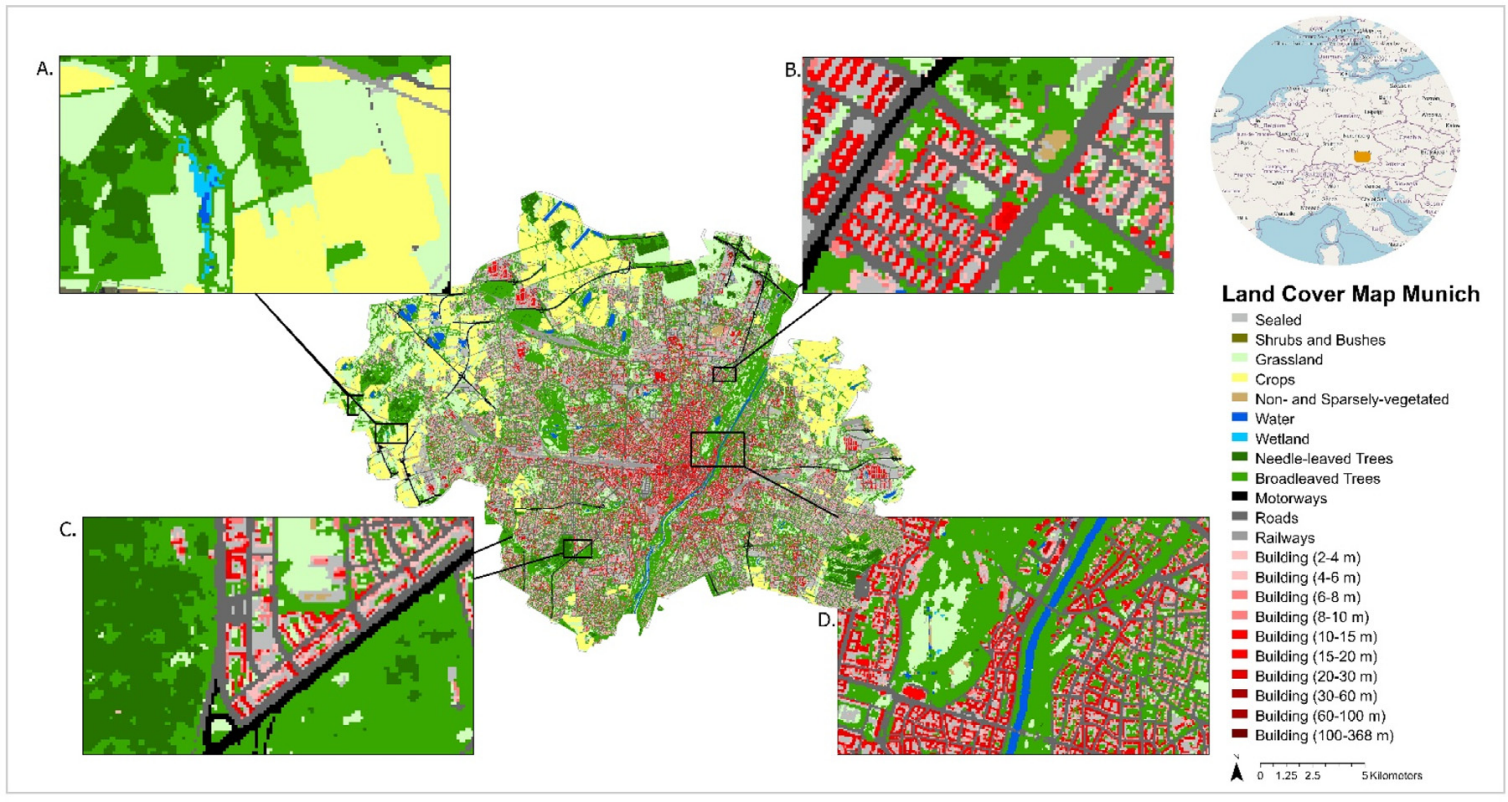
Land cover map of Munich with insets created from Copernicus data applying the described datasets and processing pipeline.

We successfully applied our described pipeline and created a land cover map for the municipality of Munich. This land cover map entails nearly all the land cover classes assembled from the Copernicus datasets with the building heights encompassing 10 height classes (Fig. 5). Only the evergreen deciduous forest and the class lichens and mosses are not represented since this vegetation does not occur in the temperate climate of Munich.

The land cover map details a high building density in the city centre of Munich with the highest building heights concentrated there. Deciduous and coniferous trees are mapped along the Isar river that traverses Munich in a north-south direction (Fig. 5d), as well as in forests surrounding the city (Fig. 5a and 5ca), but one can also spot urban trees as single pixels between the sealed, road, and building cells in the city centre (Fig. 5d). Entering the city from the West and leading to the main station in the city centre, the most prominent train connection to Munich's main station is visible. Sealed surfaces are typically found within building blocks representing sealed back and front yards (Fig. 5b). They are clearly distinguished from the roads surrounding the building blocks (Fig. 5b). Bushes and shrubs are quite rare, and crops concentrate at the fringes of the city. Thus, the Copernicus land cover map of Munich captures the main structure of the city (Fig. 5).

Moreover, the Copernicus map’s ability to depict land covers varying at a small spatial scale is demonstrated in the city centre where street trees can be visually differentiated from sealed and built-up surfaces. Similarly, the map can differentiate the roads used for car traffic from the sealed surfaces that experience rare car traffic but are often used by pedestrians and cyclists. The identification of smaller water bodies, for example, the small artificial lakes in the English garden, also supports that the map can locate various small features.

### Accuracy assessment

To validate our pipeline for creating a Copernicus urban land cover map, we followed the standard approach and performed an accuracy assessment with an independent dataset [10]. We used very high-resolution imagery of the area of interest as ground-truthing data and employed stratified random sampling to select points representing each land cover class [24]. To compute a confusion matrix (Table 3), we applied a blind approach. This assessment was conducted in ArcGIS Pro v3.3 using the integrated geoprocessing *Image Analyst Tool* [25]*.*

**Table 3:** Confusion matrix from accuracy assessment of established Copernicus land cover map.

Classes	Sealed	Bushes and Shrubs	Grassland	Crops	Non- and sparsely vegetated	Water	Wetland	Needle-leaved trees	Broadleaved deciduous trees	Roads and Highways	Railways	Buildings	Total	User Accuracy	Kappa
Sealed	54	0	1	0	2	0	0	0	1	6	1	2	67	0.81	0
Bushes and Shrubs	4	7	3	0	0	0	0	0	0	1	1	0	16	0.44	0
Grassland	2	0	56	0	0	0	2	0	2	2	0	1	65	0.86	0
Crops	0	2	1	45	0	0	0	0	0	0	0	0	48	0.94	0
Non- and sparsely vegetated	2	0	2	0	6	1	0	0	0	2	0	0	13	0.46	0
Water	0	0	0	0	0	9	4	0	0	0	0	0	13	0.69	0
Wetland	0	0	0	0	0	0	3	0	0	0	0	0	3	1.00	0
Needle-leaved trees	0	0	0	0	0	0	0	7	5	0	0	0	12	0.58	0
Broadleaved deciduous trees	3	1	4	0	1	0	1	3	101	7	0	0	121	0.83	0
Roads and Highways	1	0	0	0	0	0	0	0	0	57	0	1	59	0.97	0
Railways	0	0	1	0	0	0	0	0	2	0	9	0	12	0.75	0
Buildings	11	0	0	0	1	0	0	0	5	5	0	79	101	0.78	0
Total	77	10	68	45	10	10	10	10	116	80	11	83	530	0.00	0
**Producer Accuracy**	0.70	0.70	0.82	1	0.6	0.9	0.3	0.7	0.87	0.71	0.82	0.95	0.00	**0.82**	0
**Kappa**	0	0	0	0	0	0	0	0	0	0	0	0	0	0	**0.79**

An accuracy value of 82 % with <20 % omission and commission errors is considered good performance for this resolution data [9], with a kappa value of 0.79, representing an estimate of accuracy attenuating random effects [24]. This accuracy assessment suggests that the land cover map produced from our processing pipeline provides generally high reliability for urban ecological modelling, with some classes performing well and others having lower accuracy (Table 3). It has a marginally higher accuracy than what Oliveira et al. [10] obtained for their classification of urban climate zones.

The class confusion analysis is instrumental in identifying which land cover classes are frequently misclassified, providing a deeper comprehension of the map's potential limitations and land cover types of uncertainty. The land cover classes Roads and Highways, Crops and Wetlands exhibited a very high user accuracy (0.94 – 1.0), indicating they were nearly always correctly classified when predicted. Conversely, the classes Bushes and Shrubs, Non- and sparsely vegetated, Water, and Needle-leaved trees demonstrated low user accuracies (0.44 – 0.69), suggesting that they were frequently not correctly identified and confused with other land cover types. The confusion matrix reveals that misclassifications frequently occur between Bushes and Shrubs and Sealed or Grassland classes. Wetland was repeatedly identified as Water, and Needle-leaved trees were frequently confused with Broadleaved deciduous trees.

## Limitations

The presented pipeline to process Copernicus spatial datasets to create a standardized land cover map was shown to well represent the general structure of Munich and to have an accuracy comparable to other spatial datasets while aligning with the quality standards of remote sensing products. Compared to the foundation layer, *CLC+ Backbone 2021,* we could add 14 land cover classes to the initial 10 classes. While *CLC+ Backbone 2021* can differentiate eight vegetation types from sparsely vegetated areas and sealed surfaces, our additional processing substantially enriched the description of the sealed surface by including buildings of various heights and streets of different types. However, the presented methodology and the used datasets also have limitations.

First, the 10 m resolution of the Copernicus land cover map is coarser than several land cover maps used in the literature to model ecological connectivity in cities. This is because the Copernicus datasets are created from satellite imagery, which is the only source of remote sensing data available at the continental scale, whereas other studies reviewed used data obtained from airborne sensors. Thus, from currently available public datasets consisting of thematic layers, achieving higher spatial resolution is not feasible when aiming for continental coverage.

A similar issue applies to the thematic resolution of the created Copernicus land cover map. While we were able to differentiate many land cover classes and reach thematic granularity comparable to other studies, more finely resolved thematic resolution might be required for certain animals. For pollinators, for example, information on the distribution of grass is insufficient to predict their occurrence or movement because they depend on pollen and nectar whereas our grass class does not provide any information on the spatial distribution of flowers. Therefore, the application of the generated Copernicus land cover map to model the ecology of animals dependent on more specific land covers than the classes provided by our map cannot be recommended.

Moreover, the visual accuracy assessment of the Copernicus land cover map revealed that small, vegetated areas such as private gardens, grass, shrubs, and individual urban trees were sometimes misclassified due to their restricted size, representing mixed pixel issues. Instead of being recognized as distinct vegetated patches, they were frequently assigned to other land cover classes that occupied a larger portion of the 100 m² pixel. This loss of vegetation pixels was anticipated due to the adjacency and low overall vegetation coverage in highly dense built-up areas [14]. This is, in particular, a disadvantage since previous research highlights that small-vegetated patches like private gardens hold a substantial ecological value in urban areas [5].

The definition of urban areas represents another important challenge for urban ecology since definitions of urban areas vary considerably between countries. The EU functional cities approach provides a standardised and functional definition based on human population, but in the Urban Atlas dataset, spatial information is missing for a few European functional cities. Since the *Urban Atlas Digital Height Model 2012* provided essential information on building height, we decided to include it in our processing pipeline, accepting to compromise on geographic coverage consistency in favour of critical land cover information. Therefore, currently, our map provides land cover information for 785 functional cities in Europe, but a few are missing. Thus, we encourage the Copernicus program to expand the *Urban Atlas* datasets to all major EU functional cities while we leave the decision on whether to use administrative boundaries or the EU functional urban area definition for delineating the urban boundary to the user when defining their modelling study area.

It should be noted that since it would have been beyond the scope of this publication to review the entire field of ecological urban modelling, the criteria applied to assemble the spatial data stem from ecological connectivity modelling in cities as an example discipline. Regarding the thematic and spatial resolution, connectivity models have very high requirements [3]. Therefore, despite not using requirements from all urban ecological modelling disciplines, we applied very strict criteria to select the spatial data depicting both natural and anthropogenic land cover, making the land cover map valuable for modelling across a wide variety of disciplines.

Overall, we defined criteria that a land cover map needs to fulfil to serve as an input dataset for urban ecological modelling. The criteria were based on a literature mini-review demonstrating a lack of spatial data available at a sufficiently high spatial resolution (max. 10 m cell size) covering an entire continent. The literature mini-review also pointed to land covers typically included in similar modelling studies (trees, grass, shrubs, buildings, roads, highways, railways, sealed surfaces, bare soil) and that, therefore, should be reflected within a newly created land cover map. We applied those criteria to spatial raster data by the EU Copernicus program and selected the *CLC+ Backbone 2021*, the *Urban Atlas 2018 Digital Height Model*, the *Urban Atlas 2018 Roads and Rail Network,* and the *High Resolution Layer Water and Wetness* 2018 dataset as input data to create a finely resolved land cover map. We then described our processing pipeline to reclassify and combine these datasets to obtain a unified land cover map with high spatial and thematic resolution. The visual validation of the land cover map created for Munich and the performed accuracy assessment demonstrated that the produced land cover map can identify most land covers and structures within the city and that its accuracy lies in a range that is considered valid for remotely sensed spatial information. While the spatial and thematic resolution could still limit the application of this map to modelling different properties of smaller or more specialized species, the land cover classes and the spatial resolution are within the range of other studies that used regional geodata. Thus, our pan-European pipeline for land cover maps holds the opportunity to facilitate urban ecological modelling by omitting the labour-intensive process of data acquisition and pre-processing, making urban ecological modelling more comparable and thereby fostering the application of models generated in one city to other major European cities.

## Ethics statements

The work did not involve human beings.

The work did not involve animals.

The work did not involve data collected from social media platforms.

## CRediT author statement

**Meret Pundsack:** Conceptualization, Methodology, Software, Data curation, Visualization, Validation, Writing – original draft. **Lisa Merkens:** Conceptualization, Methodology, Software, Data curation, Visualization, Investigation, Writing – original draft, Supervision. **Wolfgang W. Weisser:** Conceptualization, Writing – review & editing, Supervision. **Anne Mimet:** Conceptualization, Methodology, Writing – reviewi & editing, Supervision.

## Declaration of competing interest

The authors declare that they have no known competing financial interests or personal relationships that could have appeared to influence the work reported in this paper.

## Appendix Supplementary materials

**Supplementary material**The methodology presented in this paper, the Copernicus pipeline, is available at https://gitlab.com/LMerkens/urban-connectivity-public. The linked GitLab repository includes a README file with setup instructions and code usage guidelines, as well as an R Markdown (.Rmd) file containing the code to reproduce the pipeline and to generate the land cover map results. Image, application 1
