# Supplementary material for: A Copernicus pipeline to create a highly resolved land cover map for modelling urban biodiversity in European cities
Source: MethodsX. 2025 Jun 10;15:103415. doi: 10.1016/j.mex.2025.103415 (PMC12221427; doi:10.1016/j.mex.2025.103415)
Supplement: Supplementary file 1 — Supplementary materialThe methodology presented in this paper, the Copernicus pipeline, is available at https://gitlab.com/LMerkens/urban-connectivity-public. The linked GitLab repository includes a README file with setup instructions and code usage guidelines, as well as an R Markdown (.Rmd) file containing the code to reproduce the pipeline and to generate the land cover map results. [file mmc1.docx]

**A Copernicus pipeline to create a highly resolved land cover map for modelling urban biodiversity in European cities**

**Supplementary information**

Meret Pundsack*, Lisa Merkens*, Wolfgang W. Weisser, Anne Mimet

* equal contribution

**Appendix 1: Publications included in mini review on spatial and thematic resolution of land cover maps used for ecological connectivity modelling in cities**

[1] M. App, M.W. Strohbach, A.-K. Schneider, B. Schröder, Making the case for gardens: Estimating the contribution of urban gardens to habitat provision and connectivity based on hedgehogs (Erinaceus europaeus), Landscape and Urban Planning 220 (2022) 104347. https://doi.org/10.1016/j.landurbplan.2021.104347.

[2] M. Balbi, S. Croci, E.J. Petit, A. Butet, R. Georges, L. Madec, J. Caudal, A. Ernoult, Least‐cost path analysis for urban greenways planning: A test with moths and birds across two habitats and two cities, Journal of Applied Ecology 58 (2021) 632–643. https://doi.org/10.1111/1365-2664.13800.

[3] M. Balbi, E.J. Petit, S. Croci, J. Nabucet, R. Georges, L. Madec, A. Ernoult, Ecological relevance of least cost path analysis: An easy implementation method for landscape urban planning, Journal of Environmental Management 244 (2019) 61–68. https://doi.org/10.1016/j.jenvman.2019.04.124.

[4] S. Beaujean, A.N.M. Nor, T. Brewer, J.G. Zamorano, A.C. Dumitriu, J. Harris, R. Corstanje, A multistep approach to improving connectivity and co-use of spatial ecological networks in cities, Landscape Ecol 36 (2021) 2077–2093. https://doi.org/10.1007/s10980-020-01159-6.

[5] J. Beninde, S. Feldmeier, M. Werner, D. Peroverde, U. Schulte, A. Hochkirch, M. Veith, Cityscape genetics: structural vs. functional connectivity of an urban lizard population, Mol Ecol 25 (2016) 4984–5000. https://doi.org/10.1111/mec.13810.

[6] T. Bhakti, J.C. Pena, B.B. Niebuhr, J. Sampaio, F.F. Goulart, C.S.D. Azevedo, M.C. Ribeiro, Y. Antonini, Combining land cover, animal behavior, and master plan regulations to assess landscape permeability for birds, Landscape and Urban Planning 214 (2021) 104171. https://doi.org/10.1016/j.landurbplan.2021.104171.

[7] S. Braaker, M. Moretti, R. Boesch, J. Ghazoul, M.K. Obrist, F. Bontadina, Assessing habitat connectivity for ground-dwelling animals in an urban environment, Ecological Applications 24 (2014) 1583–1595. https://doi.org/10.1890/13-1088.1.

[8] S. Braaker, U. Kormann, F. Bontadina, M.K. Obrist, Prediction of genetic connectivity in urban ecosystems by combining detailed movement data, genetic data and multi-path modelling, Landscape and Urban Planning 160 (2017) 107–114. https://doi.org/10.1016/j.landurbplan.2016.12.011.

[9] K. Driezen, F. Adriaensen, C. Rondinini, C.P. Doncaster, E. Matthysen, Evaluating least-cost model predictions with empirical dispersal data: A case-study using radiotracking data of hedgehogs (Erinaceus europaeus), Ecological Modelling 209 (2007) 314–322. https://doi.org/10.1016/j.ecolmodel.2007.07.002.

[10] M. Egerer, N. Fouch, E.C. Anderson, M. Clarke, Socio-ecological connectivity differs in magnitude and direction across urban landscapes, Sci Rep 10 (2020) 4252. https://doi.org/10.1038/s41598-020-61230-9.

[11] E. Ersoy, A. Jorgensen, P.H. Warren, Identifying multispecies connectivity corridors and the spatial pattern of the landscape, Urban Forestry & Urban Greening 40 (2019) 308–322. https://doi.org/10.1016/j.ufug.2018.08.001.

[12] M. Grabow, J.L.P. Louvrier, A. Planillo, S. Kiefer, S. Drenske, K. Börner, M. Stillfried, R. Hagen, S. Kimmig, T.M. Straka, S. Kramer-Schadt, Data-integration of opportunistic species observations into hierarchical modeling frameworks improves spatial predictions for urban red squirrels, Front. Ecol. Evol. 10 (2022) 881247. https://doi.org/10.3389/fevo.2022.881247.

[13] D.R. Grafius, R. Corstanje, G.M. Siriwardena, K.E. Plummer, J.A. Harris, A bird’s eye view: using circuit theory to study urban landscape connectivity for birds, Landscape Ecol 32 (2017) 1771–1787. https://doi.org/10.1007/s10980-017-0548-1.

[14] G.R. Graviola, M.C. Ribeiro, J.C. Pena, Reconciling humans and birds when designing ecological corridors and parks within urban landscapes, Ambio 51 (2022) 253–268. https://doi.org/10.1007/s13280-021-01551-9.

[15] Q. Han, G. Keeffe, Stepping stones: Assessing the permeability of urban greenspaces to climate-driven migration of trees, SASBE 9 (2019) 246–257. https://doi.org/10.1108/SASBE-12-2018-0065.

[16] W. Hou, L. Zhai, S. Feng, U. Walz, Restoration priority assessment of coal mining brownfields from the perspective of enhancing the connectivity of green infrastructure networks, Journal of Environmental Management 277 (2021) 111289. https://doi.org/10.1016/j.jenvman.2020.111289.

[17] X. Huang, H. Wang, L. Shan, F. Xiao, Constructing and optimizing urban ecological network in the context of rapid urbanization for improving landscape connectivity, Ecological Indicators 132 (2021) 108319. https://doi.org/10.1016/j.ecolind.2021.108319.

[18] F. Kong, D. Wang, H. Yin, I. Dronova, F. Fei, J. Chen, Y. Pu, M. Li, Coupling urban 3‐D information and circuit theory to advance the development of urban ecological networks, Conservation Biology 35 (2021) 1140–1150. https://doi.org/10.1111/cobi.13682.

[19] M. Kosma, A. Laita, R. Duflot, No net loss of connectivity: Conserving habitat networks in the context of urban expansion, Landscape and Urban Planning 239 (2023) 104847. https://doi.org/10.1016/j.landurbplan.2023.104847.

[20] A. Laforge, J. Pauwels, B. Faure, Y. Bas, C. Kerbiriou, J. Fonderflick, A. Besnard, Reducing light pollution improves connectivity for bats in urban landscapes, Landscape Ecol 34 (2019) 793–809. https://doi.org/10.1007/s10980-019-00803-0.

[21] S. LaPoint, P. Gallery, M. Wikelski, R. Kays, Animal behavior, cost-based corridor models, and real corridors, Landscape Ecol 28 (2013) 1615–1630. https://doi.org/10.1007/s10980-013-9910-0.

[22] T.S. Lee, L.A. Randall, N.L. Kahal, H.L. Kinas, V.A. Carney, H. Rudd, T.M. Baker, K. Sanderson, I.F. Creed, A. Moehrenschlager, D. Duke, A framework to identify priority wetland habitats and movement corridors for urban amphibian conservation, Ecol Sol and Evidence 3 (2022) e12139. https://doi.org/10.1002/2688-8319.12139.

[23] Y. Liu, T.-T. Huang, X. Zheng, A method of linking functional and structural connectivity analysis in urban green infrastructure network construction, Urban Ecosyst 25 (2022) 909–925. https://doi.org/10.1007/s11252-022-01201-2.

[24] S.B. Magle, D.M. Theobald, K.R. Crooks, A comparison of metrics predicting landscape connectivity for a highly interactive species along an urban gradient in Colorado, USA, Landscape Ecol 24 (2009) 267–280. https://doi.org/10.1007/s10980-008-9304-x.

[25] J. Marulli, J. Mallarach, A GIS methodology for assessing ecological connectivity: application to the Barcelona Metropolitan Area, Landscape and Urban Planning 71 (2005) 243–262. https://doi.org/10.1016/S0169-2046(04)00079-9.

[26] C. Matos, S.O. Petrovan, P.M. Wheeler, A.I. Ward, Landscape connectivity and spatial prioritization in an urbanising world: A network analysis approach for a threatened amphibian, Biological Conservation 237 (2019) 238–247. https://doi.org/10.1016/j.biocon.2019.06.035.

[27] Miao, Pan, Wang, Chen, Yan, Liu, Research on Urban Ecological Network Under the Threat of Road Networks—A Case Study of Wuhan, IJGI 8 (2019) 342. https://doi.org/10.3390/ijgi8080342.

[28] A. Mimet, C. Kerbiriou, L. Simon, J.-F. Julien, R. Raymond, Contribution of private gardens to habitat availability, connectivity and conservation of the common pipistrelle in Paris, Landscape and Urban Planning 193 (2020). https://doi.org/10.1016/j.landurbplan.2019.103671.

[29] F. Molné, G.F.A. Donati, J. Bolliger, M. Fischer, M. Maurer, P.M. Bach, Supporting the planning of urban blue-green infrastructure for biodiversity: A multi-scale prioritisation framework, Journal of Environmental Management 342 (2023) 118069. https://doi.org/10.1016/j.jenvman.2023.118069.

[30] E. Morin, P.-A. Herrault, Y. Guinard, F. Grandjean, N. Bech, The promising combination of a remote sensing approach and landscape connectivity modelling at a fine scale in urban planning, Ecological Indicators 139 (2022) 108930. https://doi.org/10.1016/j.ecolind.2022.108930.

[31] A.N.M. Nor, R. Corstanje, J.A. Harris, D.R. Grafius, G.M. Siriwardena, Ecological connectivity networks in rapidly expanding cities, Heliyon 3 (2017). https://doi.org/10.1016/j.heliyon.2017.e00325.

[32] J. Shen, W. Zhu, Z. Peng, Y. Wang, Improving landscape ecological network connectivity in urbanizing areas from dual dimensions of structure and function, Ecological Modelling 482 (2023) 110380. https://doi.org/10.1016/j.ecolmodel.2023.110380.

[33] A. Shimazaki, Y. Yamaura, M. Senzaki, Y. Yabuhara, T. Akasaka, F. Nakamura, Urban permeability for birds: An approach combining mobbing-call experiments and circuit theory, Urban Forestry & Urban Greening 19 (2016) 167–175. https://doi.org/10.1016/j.ufug.2016.06.024.

[34] Y. Tang, C. Gao, X. Wu, Urban Ecological Corridor Network Construction: An Integration of the Least Cost Path Model and the InVEST Model, IJGI 9 (2020) 33. https://doi.org/10.3390/ijgi9010033.

[35] C. Tannier, M. Bourgeois, H. Houot, J.-C. Foltête, Impact of urban developments on the functional connectivity of forested habitats: a joint contribution of advanced urban models and landscape graphs, Land Use Policy 52 (2016) 76–91. https://doi.org/10.1016/j.landusepol.2015.12.002.

[36] S. Tarabon, C. Calvet, V. Delbar, T. Dutoit, F. Isselin-Nondedeu, Integrating a landscape connectivity approach into mitigation hierarchy planning by anticipating urban dynamics, Landscape and Urban Planning 202 (2020) 103871. https://doi.org/10.1016/j.landurbplan.2020.103871.

[37] G. Verbeylen, L. De Bruyn, F. Adriaensen, E. Matthysen, Does matrix resistance influence Red squirrel (Sciurus vulgaris L. 1758) distribution in an urban landscape?, Landscape Ecology 18 (2003) 791–805. https://doi.org/10.1023/B:LAND.0000014492.50765.05.

[38] D. Yu, B. Xun, P. Shi, H. Shao, Y. Liu, Ecological restoration planning based on connectivity in an urban area, Ecological Engineering 46 (2012) 24–33. https://doi.org/10.1016/j.ecoleng.2012.04.033.

[39] A. Zetterberg, U.M. Mörtberg, B. Balfors, Making graph theory operational for landscape ecological assessments, planning, and design, Landscape and Urban Planning 95 (2010) 181–191. https://doi.org/10.1016/j.landurbplan.2010.01.002.

[40] M. Zhang, J. Li, L. Wang, B. Xu, W. Nie, The impact of connectivity in natural protected areas on the resilience of urban ecological networks: A research framework based on hierarchical disturbance scenario simulation, Ecological Indicators 164 (2024) 112144. https://doi.org/10.1016/j.ecolind.2024.112144.

[41] S. Zhao, Y. Ma, J. Wang, X. You, Landscape pattern analysis and ecological network planning of Tianjin City, Urban Forestry & Urban Greening 46 (2019) 126479. https://doi.org/10.1016/j.ufug.2019.126479.
